# Cediranib in addition to chemotherapy for women with relapsed platinum-sensitive ovarian cancer (ICON6): overall survival results of a phase III randomised trial

**DOI:** 10.1016/j.esmoop.2020.100043

**Published:** 2021-02-18

**Authors:** J.A. Ledermann, A.C. Embleton-Thirsk, T.J. Perren, G.C. Jayson, G.J.S. Rustin, S.B. Kaye, H. Hirte, A. Oza, M. Vaughan, M. Friedlander, A. González-Martín, E. Deane, B. Popoola, L. Farrelly, A.M. Swart, R.S. Kaplan, M.K.B. Parmar, ICON6 collaborators

**Affiliations:** 1 UCL Cancer Institute, Cancer Research UK & UCL Trials Centre, London, UK; 2 UCL Comprehensive Clinical Trials Unit, London, UK; 3 Leeds Institute of Medical Research at St James's, Leeds, UK; 4 Christie Hospital and University of Manchester, Manchester, UK; 5 Mount Vernon Cancer Centre, London, UK; 6 Royal Marsden Hospital, London, UK; 7 Juravinski Cancer Centre, Hamilton, Canada; 8 Princess Margaret Cancer Centre, Toronto, Canada; 9 Christchurch Hospital, Christchurch, New Zealand; 10 Prince of Wales Clinical School, University of New South Wales, Sydney, Australia; 11 Clínica Universidad de Navarra, Madrid, Spain; 12 Medical Research Council Clinical Trials Unit at UCL, London, UK; 13 University of East Anglia, Norwich, UK

**Keywords:** oncology, ovarian, clinical trial, phase III, overall survival

## Abstract

**Background:**

Cediranib, an oral anti-angiogenic VEGFR 1-3 inhibitor, was studied at a daily dose of 20 mg in combination with platinum-based chemotherapy and as maintenance in a randomised trial in patients with first relapse of ‘platinum-sensitive’ ovarian cancer and has been shown to improve progression-free survival (PFS).

**Patients and methods:**

ICON6 (NCT00532194) was an international three-arm, double-blind, placebo-controlled randomised trial. Between December 2007 and December 2011, 456 women were randomised, using stratification, to receive either chemotherapy with placebo throughout (arm A, reference); chemotherapy with concurrent cediranib, followed by maintenance placebo (arm B, concurrent); or chemotherapy with concurrent cediranib, followed by maintenance cediranib (arm C, maintenance). Due to an enforced redesign of the trial in September 2011, the primary endpoint became PFS between arms A and C which we have previously published, and the overall survival (OS) was defined as a secondary endpoint, which is reported here.

**Results:**

After a median follow-up of 25.6 months, strong evidence of an effect of concurrent plus maintenance cediranib on PFS was observed [hazard ratio (HR) 0.56, 95% confidence interval (CI) 0.44-0.72, *P* < 0.0001]. In this final update of the survival analysis, 90% of patients have died. There was a 7.4-month difference in median survival and an HR of 0.86 (95% CI: 0.67-1.11, *P* = 0.24) in favour of arm C. There was strong evidence of a departure from the assumption of non-proportionality using the Grambsch–Therneau test (*P* = 0.0031), making the HR difficult to interpret. Consequently, the restricted mean survival time (RMST) was used and the estimated difference over 6 years by the RMST was 4.8 months (95% CI: −0.09 to 9.74 months).

**Conclusions:**

Although a statistically significant difference in time to progression was seen, the enforced curtailment in recruitment meant that the secondary analysis of OS was underpowered. The relative reduction in the risk of death of 14% risk of death was not conventionally statistically significant, but this improvement and the increase in the mean survival time in this analysis suggest that cediranib may have worthwhile activity in the treatment of recurrent ovarian cancer and that further research should be undertaken.

## Highlights

• Cediranib is an oral VEGF receptor kinase inhibitor and ICON6 explored its activity in recurrent ovarian cancer.
• In a randomised trial cediranib with chemotherapy and as maintenance significantly prolonged PFS.
• Cessation of cediranib supply led to a reduction in sample size and OS was underpowered.
• A mature survival analysis showed a non-significant increase in the median and Restricted Mean Survival Time (RMST) with cediranib.
• There was a clinical benefit of cediranib but further research is needed to determine its role in recurrent ovarian cancer.

## Introduction

Ovarian cancer is the leading cause of death from gynaecological tumours in high-income countries. Most women present with advanced disease and in more than 75% of these patients the disease recurs after frontline treatment. Many have disease that will respond again to treatment, usually with platinum-based chemotherapy, but the response duration becomes shorter with each subsequent therapy, and the disease in these patients is almost invariably fatal.^1^ Combining chemotherapies with targeted therapies to enhance their effect and/or using such drugs as maintenance therapy to improve the effectiveness of treatment by lengthening the time to disease progression and subsequent treatment, and ultimately improving survival, is a major research strategy in ovarian cancer. One approach is to inhibit angiogenesis, the process of new blood vessel formation, which is required for tumour growth.^2^ This has been validated through randomised phase III trials using the monoclonal anti-vascular endothelial growth factor (VEGF) antibody, bevacizumab, which increases the response rate to chemotherapy^3^, ^4^, ^5^ and extends progression-free survival (PFS).^3^, ^4^, ^5^, ^6^, ^7^

Cediranib (AZD2171) is an oral VEGF receptor (VEGFR 1-3) and c-Kit^8^ inhibitor that has shown antitumor activity in recurrent ovarian, colorectal, advanced biliary tract, renal and lung cancers, glioblastoma and alveolar soft-part sarcoma.^9^, ^10^, ^11^, ^12^, ^13^, ^14^, ^15^ On the basis of phase II activity in ovarian cancer^9^ we investigated the potential benefit obtained by administering cediranib together with chemotherapy and as maintenance therapy in patients with ‘platinum-sensitive’ ovarian cancer who had had radiological evidence of recurrence more than 6 months after completion of first-line chemotherapy.^16^ The ICON (International Collaboration for Ovarian Neoplasia) 6 trial was an investigator-initiated, academically-led trial (NCT00532194) developed through the GCIG (Gynecologic Cancer InterGroup), and led by the MRC Clinical Trials Unit at University College London (UCL).

The ICON6 trial opened in December 2007. In October 2011, when 380 patients had been randomised, AstraZeneca announced a cessation in the manufacture of cediranib due to disappointing results from trials in colorectal cancer, non-small-cell lung carcinoma and glioblastoma.^13^^,^^17^^,^^18^ Due to a limited drug supply the original plan to recruit 2000 patients had to be modified and reduced to 440 patients on the 20 mg dose. In order to recapture as much statistical power as possible, the primary endpoint was changed from OS to PFS. These changes were all made blind to any accumulating data from ICON6 itself. In 2016 the primary trial outcome measure was reported, demonstrating a median PFS of 11.0 months with concurrent plus maintenance cediranib in comparison to 8.7 months in the placebo arm (HR 0·56, 0.44-0.72, *P* < 0.0001). Here we report the overall survival (OS) after extended follow-up and exploratory endpoints. Thirty patients were treated at the higher 30 mg dose, but the primary analysis and this OS update focus on the 20 mg dose.^19^

## Patients and methods

### Participants and trial design

**Figure 1:**
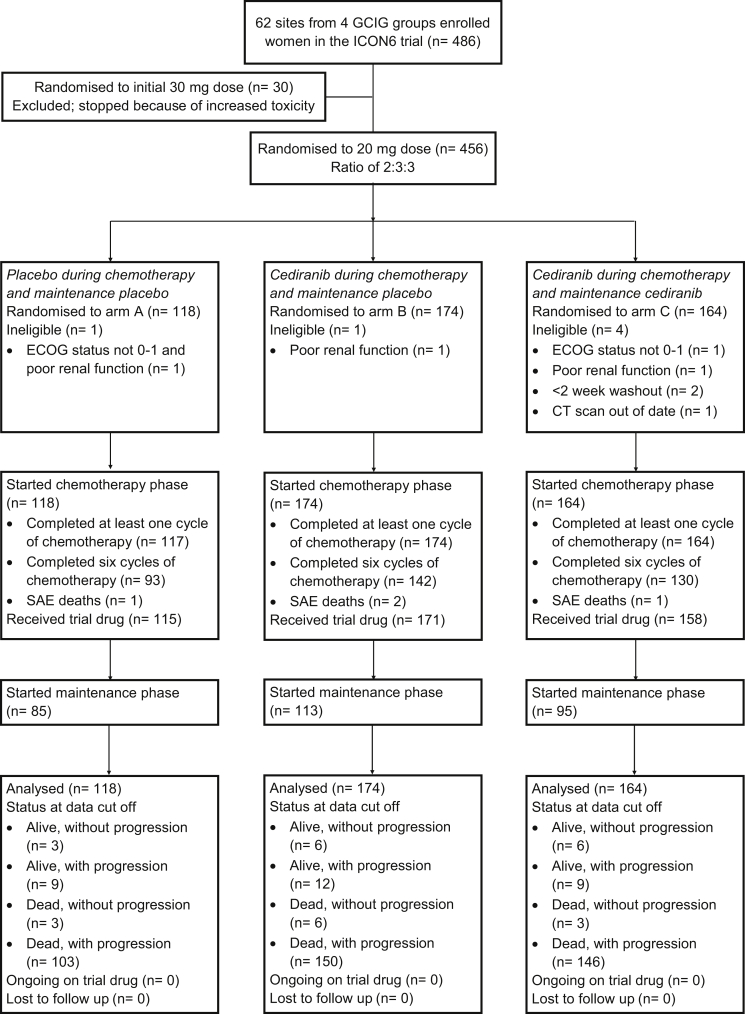
CONSORT diagram. ECOG, Eastern Cooperative Oncology Group; GCIG, Gynecologic Cancer InterGroup; SAE, serious adverse events.

Women from 62 centres in the UK, Canada, Australia, New Zealand and Spain with recurrent ovarian, fallopian tube or primary peritoneal cancer requiring further platinum-based chemotherapy ≥6 months after completing first-line chemotherapy were entered into ICON6. The inclusion and exclusion criteria have been previously described.^16^ Patients, using permuted blocks (alternating between 8 and 16) and stratified for GCIG group, first-line chemotherapy including paclitaxel, relapse-free interval (6-12/>12 months), planned chemotherapy regimen and previous bevacizumab treatment were randomised to one of three treatment arms in a 2:3:3 ratio. Arm A (reference) received chemotherapy plus daily oral placebo tablets throughout chemotherapy and continued as maintenance, arm B (concurrent) received daily oral cediranib during chemotherapy then switched to placebo during maintenance and arm C (concurrent + maintenance) received cediranib during chemotherapy and continued cediranib as maintenance. See Figure 1 for CONSORT diagram.

### Treatment

Six cycles of three-weekly chemotherapy were planned, but the maintenance phase could begin after a minimum of four cycles if patients were unable to complete six cycles. Carboplatin with either paclitaxel or gemcitabine was the recommended treatment but carboplatin monotherapy or cisplatin, if combination regimens could not be given, were allowed.^20^^,^^21^ Protocol-defined dose reductions of chemotherapy were carried out if necessary.

Trial drug, cediranib or placebo, was started with chemotherapy and continued to progression or unacceptable toxicity. An initial safety phase employed a single daily dose of 30 mg. Following review of the first 30 patients in November 2008,^19^ the Independent Data Monitoring Committee recommended reducing the dose of cediranib from 30 mg to 20 mg, in line with ongoing combination phase III trials.^10^^,^^22^^,^^23^ Clinicians were provided with clinical guidelines to aid early management of the main toxicities of cediranib which were hypertension, diarrhoea, proteinuria and fatigue. Toxicity-related interruption of trial drug for up to 2 weeks was permitted to allow recovery to grade ≤1. A dose reduction to 15 mg was permitted and required for ≥grade 3 toxicity. Cediranib/placebo was discontinued permanently if gastrointestinal perforation, arterial thromboembolic event, grade 4 haemorrhage, hypertensive crisis or reversible posterior leukoencephalopathy syndrome (RPLS) occurred.

### Statistical analysis

The revised sample size, fully described in the original publication, required 440 patients to be randomised to the 20 mg dose. In order to maximise the limited power, the primary comparison was revised from the two pairwise arm A versus arm B versus arm C comparisons to a single pairwise arm A versus arm C comparison. This decision was taken as it was considered the most appropriate comparison to target given the emerging data for concurrent and maintenance bevacizumab.^3^^,^^4^^,^^6^ The changes of primary outcome and sample size were made before any interim analysis was carried out of efficacy outcome measures. After 176 events there would be least 80% power to detect a PFS hazard ratio (HR) of 0.65 with a 5% two-sided significance level. This is detailed further in the Statistical Analysis Plan (Supplementary Material, available at https://doi.org/10.1016/j.esmoop.2020.100043) and the protocol is available at http://www.icon6.org/protocol.

At the time of the primary outcome reported in the *Lancet* by Ledermann et al.^16^, an immature analysis of OS was presented. This analysis was after 52% of patients had died and stated that OS “will be assessed again when more than 80% of deaths have occurred”. In order to maximise the limited power available, we delayed the ultimate OS analysis until now. Late in 2014, when 74% of patients had died, an unplanned analysis was carried out by AstraZeneca as part of their submission of the data to the European Medicines Agency (EMA). This analysis was eventually published online by the EMA on 21 December 2016 and “suggested a benefit in OS for [arm C] (median OS 27.9 months) compared with [arm A] (median 19.9 months). Nevertheless, this improvement was not conventionally statistically significant”.^24^ The analysis presented here was carried out with 90% of deaths having occurred in the two primary comparison arms

The revised Statistical Analysis Plan did not specify when the secondary outcome measure of OS would be analysed. However, given the reduced power, the analysis was delayed until fewer than 10% of patients were still alive. Following the reporting of the primary endpoint, the log-rank (LR) test was used as the primary test of an overall difference between Kaplan–Meier curves. A pre-specified plan to address the proportionality of hazards was made, due to the difficulty of interpreting the HR in the presence of time-dependent treatment effects. The presence of non-proportional hazards was to be assessed using the Grambsch–Therneau test.^25^ If there were evidence of non-proportionality at the 5% level, survival data would be modelled by a flexible parametric model and differences in restricted mean survival time (RMST) would be estimated, otherwise a standard Cox model would be used.^26^ Without evidence of non-proportional hazards and given the presence of non-proportional hazards in the primary endpoint of PFS, and the corresponding reduction in power when the LR test is used in its presence, further statistical methods were sought that would protect the (already severely reduced) power available to detect a difference in OS. The decision was made to utilise the Royston–Parmar combined test as well as the LR test.^27^ In a reconstruction of 50 published trials, the combined test outperformed the Cox test overall.^28^ This was implemented in Stata 15.1.^29^ OS was defined as time from randomisation to date of death from any cause. Patients who were still alive at the time of analysis were censored at the date last known to be alive.

In addition, the time to first subsequent treatment (TFST) was measured as an exploratory outcome given its emerging clinical relevance and the fact that patients could continue trial drug beyond documented disease progression.

## Results

### Overall survival

As reported previously, all three treatment groups were balanced with respect to their pre-treatment demographic characteristics.^16^ The 456 women receiving cediranib 20 mg/placebo had a median age of 62 years and were randomised at a median of 19.6 months from first diagnosis. No patients were lost to follow-up; five withdrew consent for collection of further data.

**Figure 2:**
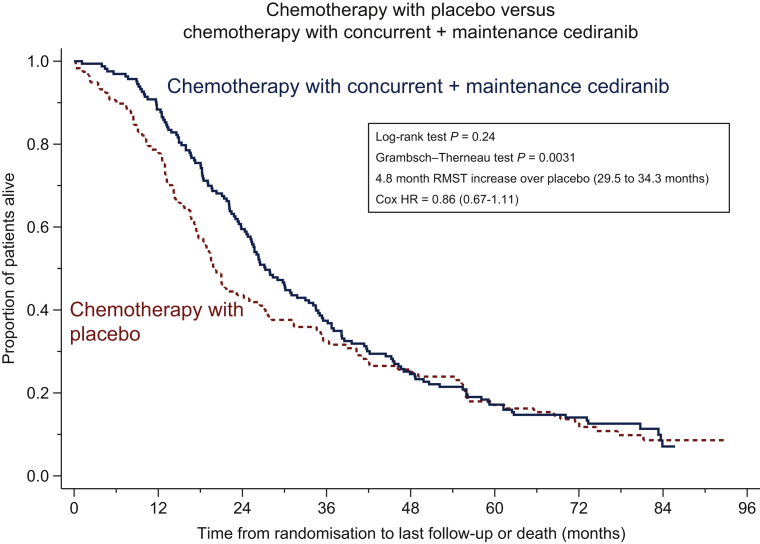
Overall survival. Kaplan–Meier plot of overall survival: (a) Arms A versus C and (b) arms A versus B versus C (number at risk every 6 months with the number of failure events in parentheses, after the time in which the number at risk was calculated). HR, hazard ratio; RMST, restricted mean survival time.

Median follow-up, using the inverse Kaplan–Meier method, was 7 years at 83.7 months in arms A and C with a database cut-off date of 1 October 2018. At this point 106/118 (90%) deaths in arm A and 149/164 (91%) in arm C had occurred. The median survival in arm A was 19.9 months [95% confidence interval (CI): 17.4-26.5] and in arm C 27.3 months (24.8-33.0), a difference of 7.4 months. The HR estimate was 0.86 with a 95% CI of 0.67-1.11. The survival differences in the Kaplan–Meier plot (Figure 2) appear to show a separation until around 30 months. Using the LR test, the difference in survival was not conventionally statistically significant (*P* = 0.24), but there was strong evidence of a departure from the assumption of non-proportionality using the Grambsch–Therneau test, *P* = 0.0031.^25^ Consequently, we calculated the RMST as a measure of the difference in OS. Concurrent and maintenance treatment with cediranib (arm C) was associated with increased time until death over a period of 6 years by a mean of 4.8 (95% CI: −0.09 to 9.74) months over the reference (arm A), from 29.5 to 34.3 months. A similar number of deaths occurred (90%:156/174) in arm B (concurrent treatment) and the median survival time was closer to the concurrent + maintenance arm (arm C) at 26.6 months (RMST 32.0 months), again consistent with the suggested positive effect of cediranib (Figure 2). Results for all 486 patients randomised, including those on the 30 mg dose, are consistent with this result and are included in the Supplementary Material, available at https://doi.org/10.1016/j.esmoop.2020.100043.

**Figure 3:**
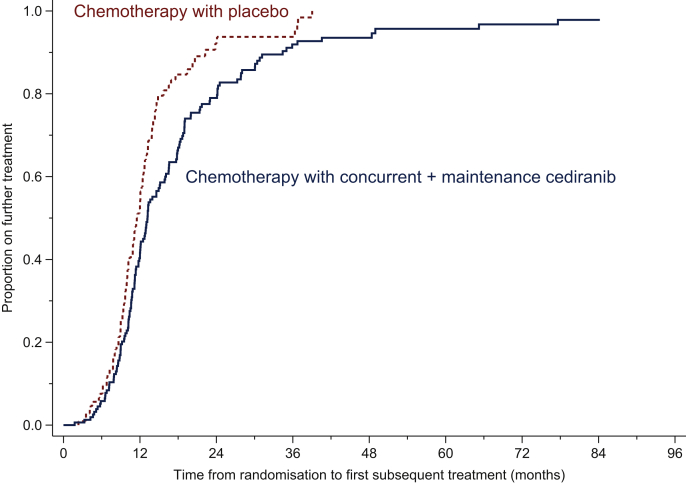
Time from randomisation to first subsequent treatment. Kaplan–Meier plot of the time from randomisation to patients' first subsequent treatment in months.

Cediranib as a maintenance treatment prolonged the median TFST compared with placebo with a difference in RMST of 5.2 months from 13.2 to 18.5 months. An HR of 0.64 (0.49-0.85) was observed, illustrating that patients on cediranib were delayed going onto a subsequent treatment, LR *P* value = 0.0014 (Figure 3). Cediranib during chemotherapy alone (arm B) did not lengthen TFST, with a median interval of 11.7 months.

### Toxicity

No additional toxicity has been reported since the detailed primary results of the primary outcome, reported in 2016. The median time on trial drug for arm A was 8.2 months and arm C, 7.6 months. A significant number of patients continued on trial drug beyond 12 months, noticeably in the maintenance arm compared with placebo (26% versus 19%) and this difference persisted at 18 months (11% versus 5%). One patient on the maintenance arm continued cediranib treatment for 5.4 years before developing progressive disease. Three patients (2.5%) on arm A and six patients (3.7%) on arm C never started any trial treatment.

In those patients who received trial drug for at least 12 months [22 (19%) in arm A and 42 (26%) in arm C], and concentrating on the toxicity events occurring after 12 months, we observed an increased incidence of the four most common side-effects in patients taking cediranib. Grade 3 fatigue occurred in 5% of patients (one patient) in arm A but in 14% (six patients) in arm C. Grade 2 diarrhoea was reported by 14% (three) of patients in arm A but by 50% (21 patients) in arm C. Grade 3 diarrhoea was also more common in patients on cediranib compared with placebo, with 5% (one patient) in arm A versus 24% (10 patients) in arm C. Hypertension generally was more common; at grade 2.18% (four patients) versus 38% (16 patients) and grade 3.5% (1 patient) versus 10% (4 patients). Grade 2 nausea was less common in arm A (four patients, 18%) than arm C (11 patients, 26%). This slight increase was also mirrored in grade 3 nausea with 5% (1 patient) versus 10% (four) for patients on cediranib.

The only incidents of grade 4 or above were experienced by <5% of patients; these were one grade 4 thrombosis and one intra-operative injury, both in arm A.

Of the 26% (42/164) of patients in arm C who were on trial drug at 1 year, 12% (5/42) stopped due to toxicity rather than progression.

## Discussion

We report the final survival analysis of women receiving platinum-based chemotherapy with or without placebo-controlled concurrent or concurrent/maintenance cediranib for platinum-sensitive ovarian cancer treated at first relapse. Although the primary analysis of ICON6 showed a significant improvement in PFS with cediranib, this did not lead to a statistically significant survival benefit. This interim comparison of cediranib with chemotherapy and as maintenance reported a 5.3-month median difference in OS. Cediranib or placebo was taken for a median of 8.3 (95% CI: 7.5-9.2) months in Arm B and 8.5 (95% CI: 6.4-9.8) in arm C.^16^ In this update we observed a median OS of 27.3 months (95% CI: 24.8-33.0) in arm C and 19.9 months (95% CI: 17.4-26.5) in arm A and HR of 0.86 (95% CI 0.67-1.11). Several factors need to be taken into consideration when interpreting these results. Firstly, the trial was initially planned as a large randomised trial with OS as the primary outcome. ICON6 became a much smaller study due to a restriction of the supply of the drug resulting in implementation of PFS as the primary endpoint. Demonstrating an OS advantage in a small trial where 90% of patients have post-progression treatment and a median post-progression survival of 14.4 months is very unlikely indeed. Secondly, interpretation of the HR and comparisons of median outcome intervals are less reliable in situations where there is non-proportionality of the survival curves. Consequently we have presented restricted median survival times, showing an increased time until death over a period of 6 years by a mean of 4.8 (95% CI: −0.09 to 9.74) months over placebo, from 29.5 to 34.3 months.

Before the definitive OS analysis reported here, a second interim OS analysis occurred following a request for an update from the Committee for Medicinal Products for Human Use (CHMP) at the EMA in November 2014. The updated analysis for the EMA took place after 74% of deaths had occurred with a data cut-off of 1 November 2014. The HR at this point in time was 0.84 (95% CI: 0.63-1.11).^24^ In preparation for this, three processes were put in place to refine the data, using the same data cut-off. These were source data verification, quality checking of data entry, and blinded independent central review of scans. All had only a limited impact on the original reported PFS results.^30^ The company ultimately withdrew their application on 9 September 2016 (13 months later).^31^

The clinical behaviour of the vascular targeting drug, cediranib, may be similar to that of other anti-angiogenic drugs, notably bevacizumab, which when given with chemotherapy and then as maintenance demonstrated improvements in PFS without significant OS gains in first-line or recurrent ovarian cancer treatment.^3^^,^^4^^,^^6^^,^^7^ Nevertheless, such improvements in PFS are considered clinically meaningful and bevacizumab is licensed for use in first-line therapy and treatment of recurrent ovarian cancer. In recurrent ovarian cancer the mature survival analysis in the OCEANS trial with bevacizumab and chemotherapy has shown no separation of the treatment and control arms when reported with >70% deaths.^32^^,^^33^ In the OCEANS trial there was a 1.7-month median OS difference between bevacizumab and placebo.^32^ A larger difference in median OS of 5.9 months was seen in GOG-0213 in which bevacizumab was added to carboplatin and paclitaxel. The analysis of GOG-0213 was carried out when 62% of patients had died and the median OS was 42.2 months (95% CI 37.7-46.2) versus 37.3 months (32.6-39.7) in the chemotherapy group (HR 0.829; 95% CI 0.683-1.005; *P* = 0.056).^7^ However, for cediranib, with 90% deaths, the analysis shows that there is an increase of 7.4 months in the median OS and 4.8 months in the RMST over 6 years. At 18 months, 6% more patients were on cediranib (11%) than on placebo (5%) as shown in Table 1.

**Table 1:** Patients' time on treatment

	Arm A (118)	Arm B (174)	Arm C (164)
On treatment >12 months	22 (19%)	31 (18%)	42 (26%)
On treatment >18 months	6 (5%)	13 (7%)	18 (11%)
On treatment >24 months	3 (3%)	4 (2%)	5 (3%)
On treatment >48 months	0	0	1 (<1%)
Median time on treatment	8.2 months	8.2 months	7.6 months
Maximum time on treatment	3.0 years	3.3 years	5.4 years

The OS of patients in the ICON6 placebo arm was 19.9 months. This is lower than other comparable trials using platinum-based combination therapy for recurrent ovarian cancer. In the OCEANS trial the median OS for the placebo arm was 35.2 months and in the CALYPSO trial it was 30.7 months and 33.0 months in the chemotherapy arms with carboplatin and either liposomal doxorubicin or paclitaxel.^4^^,^^21^^,^^34^ However, a recent analysis of survival of 4739 patients following relapse from randomised first-line phase III trials through the GOG group showed a median survival of 21.4 months and a median of 25.4 months when the most recent trial GOG-0218 is included, suggesting patient selection may have an influence on outcome.^35^ We considered whether the lower median survival in ICON6 could be due to (i) a short time to progression, (ii) a reduced post-progression survival or (iii) differences in the start of relapse therapy from the time of diagnosis. The PFS in arm A (placebo) was 8.4 months. It was 10.4, 11.3 and 9.4 months in GOG-0213, OCEANS and CALYPSO, respectively.

Ninety per cent of patients participating in ICON6 underwent third-line treatment; a similar percentage of post-progression treatments to that reported in the OCEANS trial (88.8% and 91.3% patients on the bevacizumab and placebo arms respectively, and also in the CALYPSO trial^34^). Post-hoc, estimating the overall survival in ICON6 from the date of histological diagnosis, the median OS was 43.3 and 51.3 months for control and cediranib patients (arms A and C, see Supplementary Material, available at https://doi.org/10.1016/j.esmoop.2020.100043), respectively, resembling the differences seen with placebo or bevacizumab in GOG-0218, which were 41.1 and 43.4 months, respectively.^33^ The ICON6 trial was conducted, like the OCEANS and GOG-0213 trials, before BRCA testing was carried out and the use of PARP inhibitors became widespread. Thus, the outcome of patients enrolled in the ICON6 trial was broadly similar to a cross-section of GOG-led trials in ovarian cancer.^35^

Whilst OS remains the most powerful endpoint in evaluating a new anti-cancer therapy, there is increasing recognition that drugs that demonstrate improvements in PFS and other clinically relevant endpoints can be useful additions to the therapeutic options available to patients even in the absence of an OS benefit. In this regard, TFST is increasingly recognised as a useful endpoint.^36^ This is particularly the case for maintenance drugs which by definition are intended to maintain a stabilised disease state, postponing introduction of a further chemotherapy regimen. For cediranib the difference in the median time from randomisation to the start of a further line of treatment or death was 1.7 months, from 11.5 to 13.2 months.

All of the published trials of oral VEGFR inhibitors in ovarian cancer have shown a benefit in PFS, yet none has been licensed for treatment of recurrent ovarian cancer.^37^^,^^38^ However, the positive PFS results from the ICON6 trial have led to a resumption in the manufacture of the drug and further trials in recurrent ovarian cancer. Different approaches are being studied by NRG Oncology (USA) and the NCRI Group (UK). Initial studies suggested a synergy combining cediranib with olaparib, a PARP inhibitor,^39^ and it has more recently been shown that cediranib supresses the DNA homologous recombination repair pathway making tumours more sensitive to the PARP inhibitor.^40^ The US National Cancer Institute (NCI) have tested the combination of cediranib and olaparib in a randomised trial, comparing it to olaparib alone, or intravenous platinum-based chemotherapy in recurrent ovarian cancer (NRG-GY004, NCT02446600). The results recently reported showed that adding cediranib to olaparib improves anti-tumour activity, but the combination is not superior to chemotherapy.^41^ The ICON group is conducting a trial of maintenance cediranib and olaparib, compared with olaparib alone after platinum-based chemotherapy (ICON9, NCT03278717).

Early management of toxicity is important if patients are to remain on the drug for a prolonged period. Even so, after 12 months of therapy there was a higher incidence of fatigue, diarrhoea and hypertension compared with the placebo so that careful monitoring of patients is required. Of the 26% (42/164) of patients in arm C who were on trial drug for 1 year only, 12% (5/42) stopped treatment due to toxicity. However, in spite of these toxicities there was no detrimental effect on quality of life at 1 year.^42^ In conclusion, the non-significant improvement in survival and significant PFS benefit seen with cediranib in ICON6 support further research with this drug in the treatment of recurrent ovarian cancer.
