# Supplementary material for: Cediranib in addition to chemotherapy for women with relapsed platinum-sensitive ovarian cancer (ICON6): overall survival results of a phase III randomised trial
Source: ESMO Open. 2021 Feb 18;6(2):100043. doi: 10.1016/j.esmoop.2020.100043 (PMC7903311; doi:10.1016/j.esmoop.2020.100043)
Supplement: Supplementary Material [file mmc1.pdf]

Supplementary material

Collaborators

The ICON6 trial team would like to thank all collaborators, including:

Patients

ICON6 TMG members and staff at the MRC Clinical Trials Unit at UCL – K. Brown, E. Burnett, K. Carlton, E. Clark, A. Cook, C. Davidson, C. Díaz-Montaña, E. Eisenhauer, A. Embleton, L. Farrelly, D. Fry, A. González-Martín, C. Griffin, H. Hirte, G. Jayson, C. Jones, R. Kaplan, S. Kaye, J. Ledermann, S. Mannix, J. Martyn, R. Meyer, F. Nepote, J. Parker, M. Parmar, W. Parulekar, T. Perren, J. Petrie, S. Popoola, W. Qian, F. Raja, G. Rustin, D. Stark, AM. Swart, M. Tomiczek, S. Townsend, M. Vaughan

ICON6 IDMC members – R. Buckstein, R. Coleman (Chair), U. Menon, W. Sauerbrei

ICON6 TSC members – D. Fink, P. Johnson (Chair), R. Rudd, J. Whelan

Collaborating GCIG Groups – ANZGOG, CCTG, GEICO

Recruiting Investigators and Sites – 46 UK sites, 10 Canada, 5 Australia, 2 Spain

Recruiting Principal Investigator, Institution, and Country –

F. Azribi, James Cook University Hospital, GBR

C. Barlow, Musgrove Park Hospital, GBR

C. Barlow, Yeovil District Hospital, GBR

P. Barretina, ICO Girona, ESP

S. Begbie, NCCI Port Macquarie Base Hospital, AUS

M. Beltran, ICO Girona, ESP

R. Bhana, County Hospital, GBR

J. Biagi, Cancer Centre of Southeastern Ontario at Kingston General Hospital, CAN

S. Blagden, Hammersmith Hospital, GBR

P. Bliss, Royal Devon and Exeter Hospital, GBR

T. Bonaventura, Calvary Mater Newcastle, AUS

R. Bowen, Royal United Hospital, GBR

S. Chan, Nottingham City Hospital, GBR

S. Cheesman, Bradford Royal Infirmary, GBR

M. Crawford, Airedale General Hospital, GBR

B. Crosse, Huddersfield Royal Infirmary, GBR

G. Dark, Freeman Hospital, GBR

H. Earl, Addenbrooke's Hospital, GBR

S. Ellard, BCCA-Cancer Centre for the Southern Interior, CAN

M. Friedlander, Prince of Wales/Royal Hospital for Women, AUS

H. Gabra, Hammersmith Hospital, GBR

P. Ghatage, Tom Baker Cancer Centre, CAN

M. Gore, Royal Marsden Hospital, London, GBR

M. Hall, Wexham Park Hospital, GBR

M. Hanneman, North Devon District Hospital, GBR

A. Hindley, Royal Preston Hospital, GBR

H. Hirte, Juravinski Cancer Centre at Hamilton Health Sciences, CAN

U. Hofmann, Huddersfield Royal Infirmary, GBR

M. Hogg, Royal Blackburn Hospital, GBR

P. Hoskins, BCCA-Vancouver Cancer Centre, CAN

E. Hudson, Velindre Cancer Centre, GBR

A. Hughes, Queen Elizabeth Hospital, Gateshead, GBR

G. Jayson, Christie Hospital, GBR

S. Kaye, Royal Marsden Hospital, Sutton, GBR

S. Lau, McGill University - Department of Oncology, CAN

J. Ledermann, University College Hospital, GBR

L. Li, James Cook University Hospital, GBR

F. Lofts, St George's Hospital, GBR

R. Lord, Clatterbridge Cancer Centre, GBR

R. Lord, St Helens Hospital, GBR

S. Lupton, County Hospital, GBR

A. Michael, Royal Surrey County Hospital, GBR

J. Millar, Belfast City Hospital, GBR

A. Montes, Guy's Hospital, GBR

Y. Nagar, Queen Alexandra Hospital, GBR

H. Nam, Southend University Hospital, GBR

S. Nicum, Churchill Hospital, GBR

A. Oza, University Health Network, Princess Margaret Hospital, CAN

T. Perren, St James's University Hospital, GBR

M. Persic, Queen's Hospital, Burton Upon Trent, GBR

M. Persic, Royal Derby Hospital, GBR

M. Plante, CHUQ - Pavillion Hotel-Dieu de Quebec, CAN

C. Poole, University Hospital Coventry and Warwickshire, GBR

D. Provencher, CHUM - Hopital Notre-Dame, CAN

M. Quinn, Royal Women's Hospital, AUS

N. Reed, Beatson West of Scotland Cancer Centre, GBR

J. Rubio, Hospital Reina Sofía, ESP

G. Rustin, Mount Vernon Hospital, GBR

N. Sarwar, Southend University Hospital, GBR

K. Scatchard, North Devon District Hospital, GBR

C. Steer, Border Medical Oncology, AUS

N. Stuart, Glan Clwyd Hospital, GBR

N. Stuart, Ysbyty Gwynedd, GBR

J. Summers, Maidstone Hospital, GBR

S. Sundar, King's Mill Hospital, GBR

S. Sundar, Nottingham City Hospital, GBR

K. Swenerton, BCCA-Vancouver Cancer Centre, CAN

P. Symonds, Leicester Royal Infirmary, GBR

S. Tahir, Broomfield Hospital, GBR

J. Waters, Kent & Canterbury Hospital, GBR

J. Waters, Queen Elizabeth The Queen Mother Hospital, GBR

S. Welch, London Regional Cancer Program, CAN

Expanded details of statistical analysis methods

Using Restricted Mean Survival Time, which is the most appropriate measure of effect in the presence of non-proportional hazards, we have shown some evidence of a difference between the treatment arms. Even notwithstanding the presence of non-proportional hazards Trinquart et al. recommend that "RMST-based measures should be routinely reported in randomized trials with time-to-event outcomes." [Trinquart L, Jacot J, Conner SC, Porcher R. Comparison of Treatment Effects Measured by the Hazard Ratio and by the Ratio of Restricted Mean Survival Times in Oncology Randomized Controlled Trials. J Clin Oncol. 2016 20;34(15):1813–9.] In addition, given that the comparison of arms A and C for OS was always going to be underpowered we investigated alternative analytical methods which could account for this. In the first interim Overall Survival analysis, there was some evidence of a difference, primarily 'early on' in the timeframe. Royston and Parmar suggest a 'combined test' as an improvement on the Cox test as, even though it is reasonably robust to departures from the proportionality assumption, "its power can be severely reduced in the case of an early effect".[27] The Royston-Parmar combined test utilises a standard log-rank or Cox test with a statistic derived from the maximal squared standardised between-arm difference in time-dependent RMST. This combined test is done 'non-parametrically' using RMST 'pseudovalues'. These are jackknife quantities are "derived from the Kaplan–Meier survival function and constructed so that their arithmetic mean estimates the RMST at a given time point, *t**". The two-sided p-value=0.0035 for this combined test indicated strong evidence of a difference in the survival curves. This is shown further in the Kaplan-Meier plot below. The RMST is at its maximum difference between the two arms (3.05 months) at just over two years (25.8 months).

Time-dependent Overall Survival: (a) Kaplan-Meier curves (solid lines) and estimated survival functions (dashed lines) from a flexible parametric model, red line representing 'Chemotherapy with concurrent + maintenance cediranib' and blue line representing 'Chemotherapy with placebo'; (b) difference in survival functions; (c) hazard ratio; (d) difference in restricted mean survival time. Shaded areas are pointwise 95% confidence intervals. Estimates in panels (b), (c) and (d) are derived from a flexible parametric model.

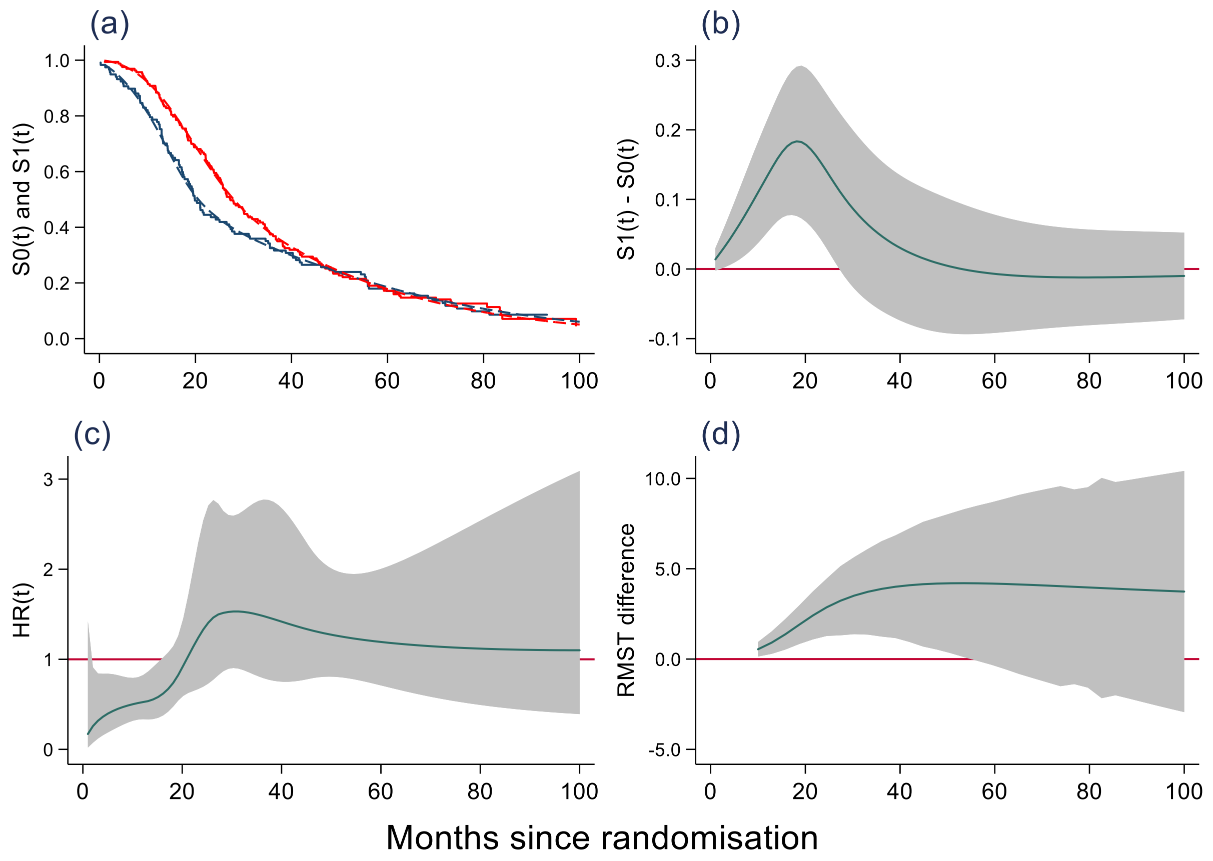

Analysis including the patients who received 30mg dose

Patients receiving the 30 mg dose:

Arm A: 7 (23%)

Arm B: 10 (33%)

Arm C: 13 (43%)

Total patients at both 20 and 30 mg doses:

Arm A: 125 (26%)

Arm B: 184 (38%)

Arm C: 177 (36%)

Median follow up= 25.7 months (22.8 – 28.35)

Log-rank test for equality of survivor functions p-value= 0.5397

Grambsch-Therneau test for non-proportionality p-value= 0.0006

RMST treatment effect estimate:

Maintenance treatment increases time until death during 6 years by 4.01 (95% CI: -1.08—9.1) months over the reference from 30.35 to 34.36 months

Cox HR estimate:

Hazard Ratio estimate 0.93 with a 95% confidence interval of (0.73 – 1.18)

Median survival (OS endpoint) times:

Reference 21.0 months (17.51 – 27.04)

Maintenance 27.9 months (25.10 – 33.48)

Difference 6.9 months

OS events/patients: 274/302 (91%)

Reference: 112/125 (90%)

Maintenance: 162/177 (92%)

Kaplan-Meier curves including the patients who received 30mg dose

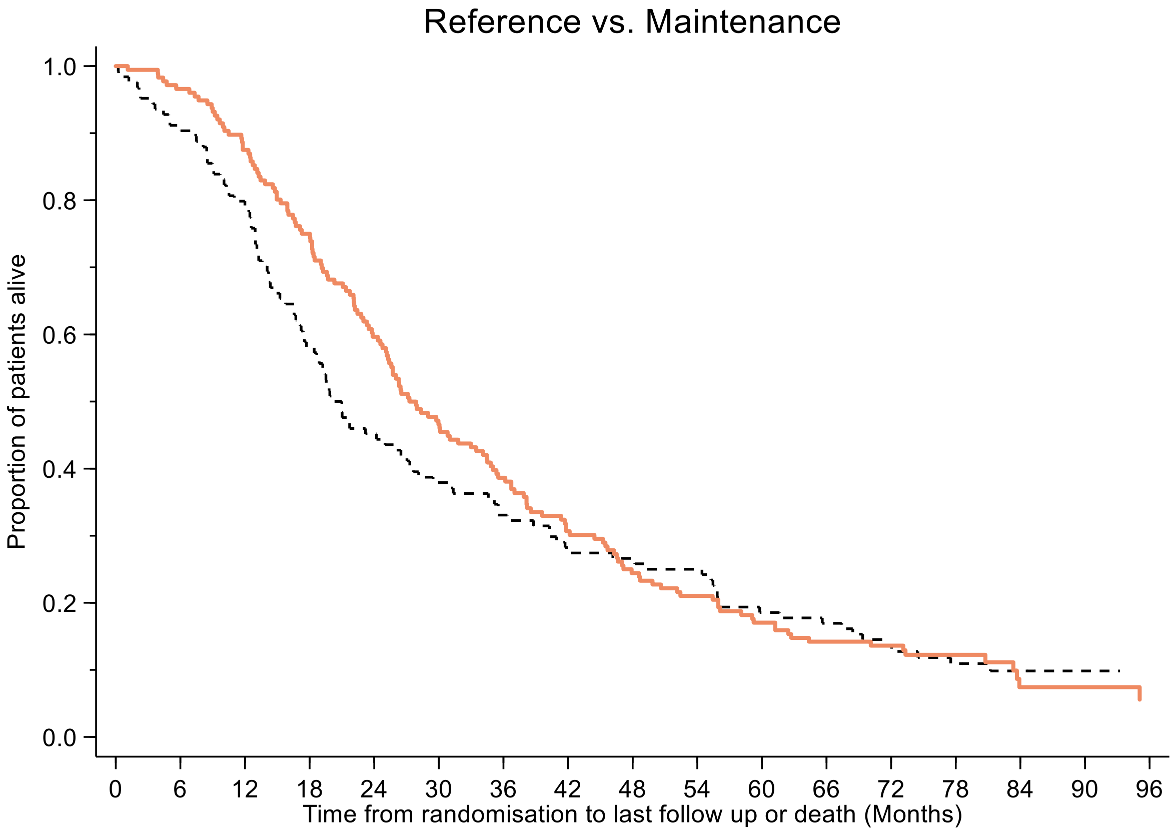

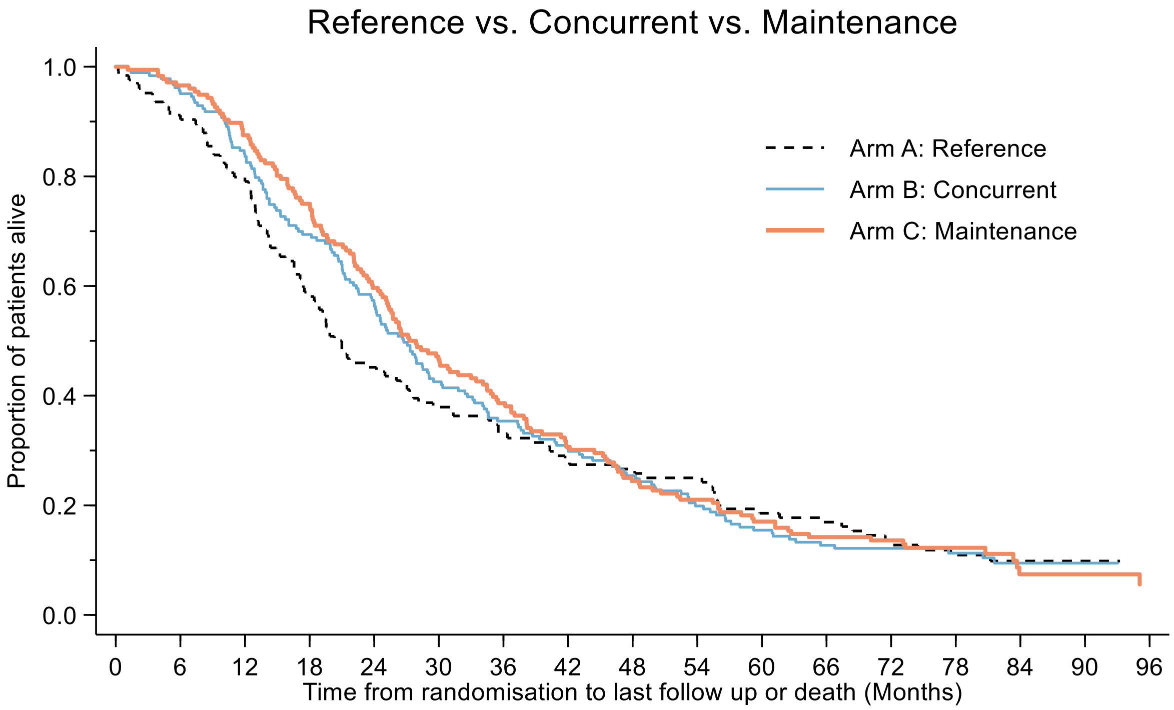

A	125	(26)	98	(42)	56	(15)	41	(8)	33	(10)	23	(6)	15	(4)	8	(0)	4
B	184	(30)	153	(49)	104	(39)	64	(18)	46	(18)	28	(6)	19	(3)	9	(0)	5
C	177	(22)	154	(49)	105	(37)	68	(25)	43	(13)	30	(6)	22	(6)	6	(1)	3

Overall Survival – time from histological diagnosis

The median time from initial diagnosis to randomisation in ICON6 was 1.6 years (IQR: 1.20—2.35), in all three arms. Adding in the trial period, the median survival time from histological diagnosis to death was an increase in median survival from 43.3 months in arm A to 51.3 months in arm C. The tests for a difference of the survival curves from histological diagnosis mirrored the time from randomisation with a log-rank test p=0.33 and a combined test p=0.02. The Hazard Ratio was 0.88 (0.69—1.14) [HR stratified by randomisation factors 0.77 (0.58—1.01)], but again, evidence of non-proportionality was present, with Gramsch-Thernau test p=0.0033. The improvement in RMST was 5.4 months from 45.9 to 51.3 months over a period of 72 months.

Kaplan-Meier plot of overall survival over nine years, number at risk every 12 months with the number of failure events in parentheses, after the time in which the number at risk was calculated.

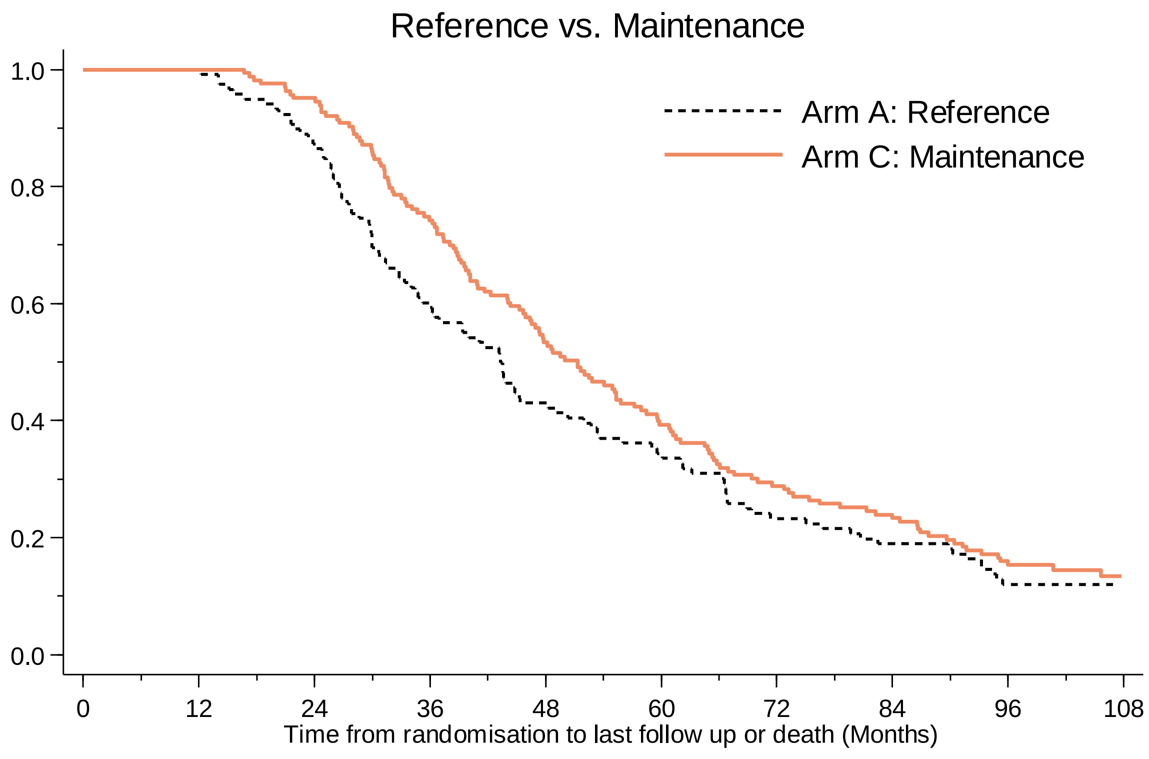

A	118	(0)	118	(15)	103	(33)	70	(19)	50	(10)	40	(13)	27	(5)	22	(8)	14	(0)	12
B	164	(0)	164	(8)	156	(34)	121	(34)	87	(23)	64	(17)	47	(9)	38	(13)	24	(2)	12

Subgroup analyses for Overall Survival by randomisation factors

Prior bevacizumab

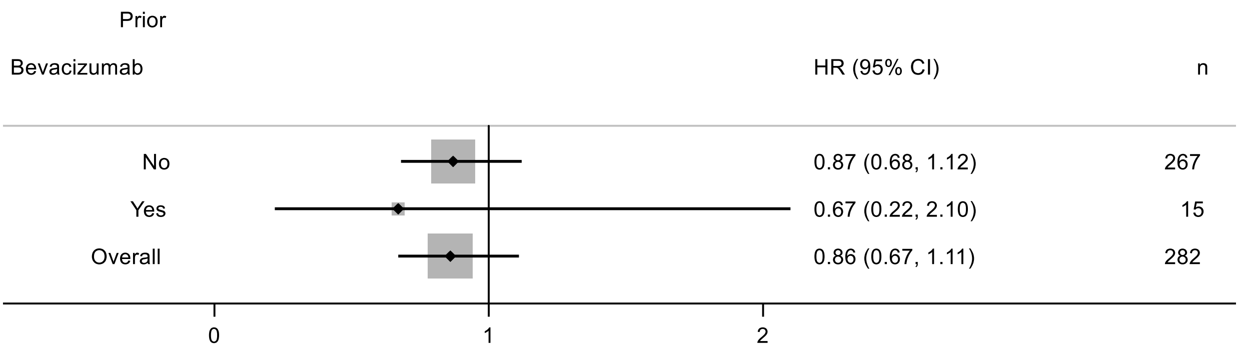

Paclitaxel

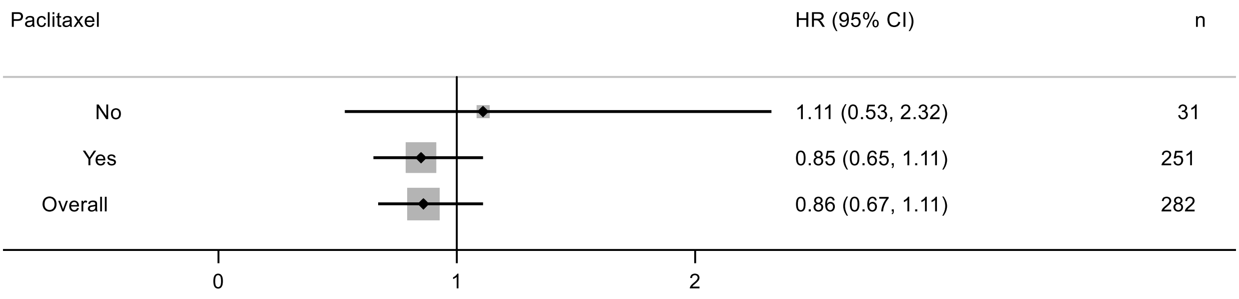
Paclitaxel
HR (95% CI)
n
No
1.11 (0.53, 2.32)
31
Yes
0.85 (0.65, 1.11)
251
Overall
0.86 (0.67, 1.11)
282
0
1
2

Planned chemotherapy

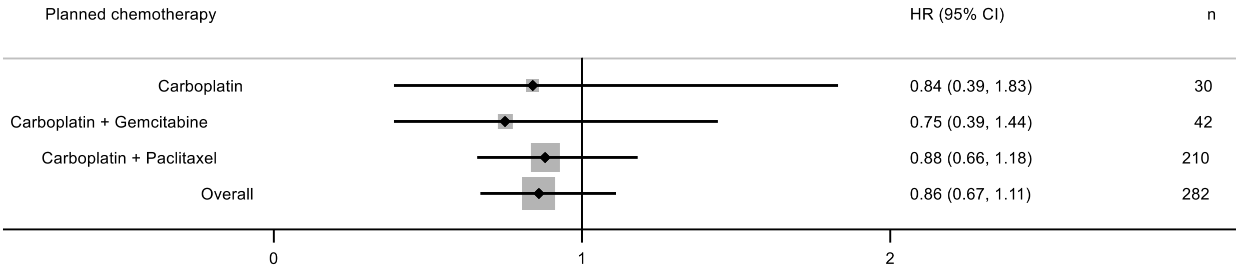

Time since last chemotherapy

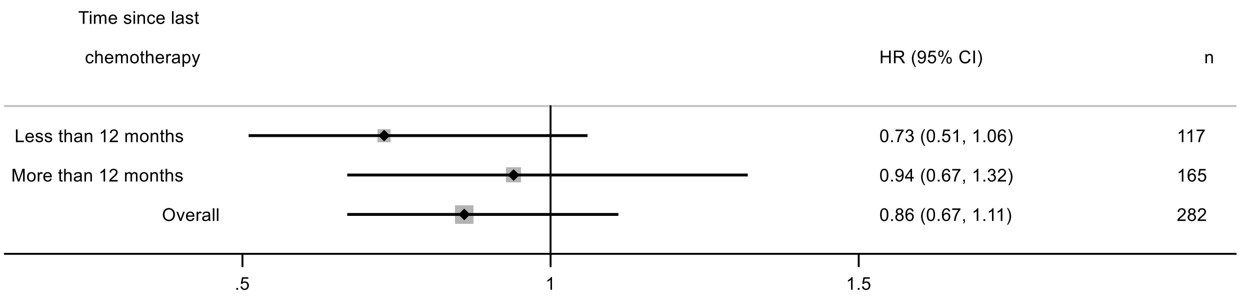

GCIG co-operative group

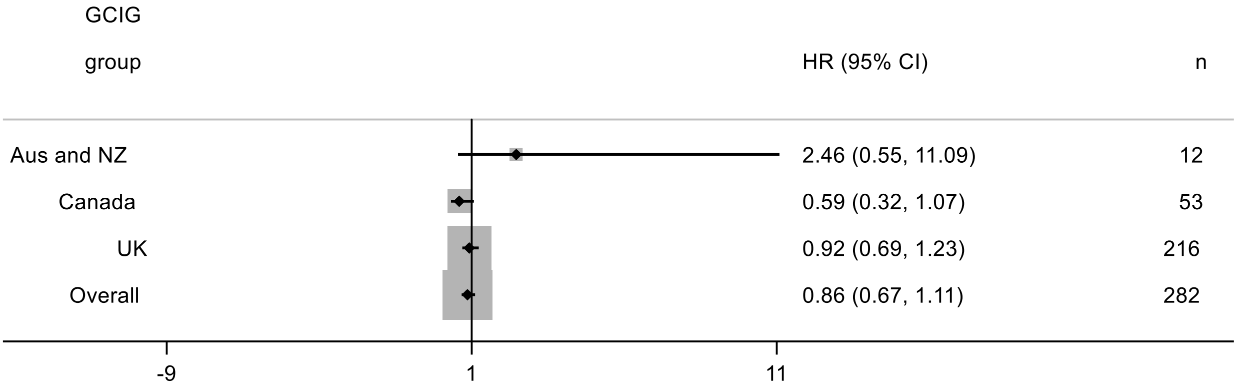

A randomised trial of concurrent cediranib [AZD2171] (with platinum-based chemotherapy) and maintenance cediranib in women with platinum-sensitive relapsed ovarian cancer

EudraCT number: 2007-001346-41

Statistical Analysis Plan

Final, version 1.0

16-Apr-2013

Version	Date	Person responsible	Comments
0.1	-	-	Summary in the Protocol
0.2	21-May-2012	Andrew Embleton	First draft
0.3	25-Jun-2012	Andrew Embleton	Updated following feedback from Fharat Raja, Mahesh Parmar and Patrick Royston
0.4	08-Aug-2012	Andrew Embleton	Updated following feedback from Mahesh Parmar
1.0	16-Apr-2013	Andrew Embleton	Finalised following feedback from Adrian Cook, Ann Marie Swart and Mahesh Parmar

**Role:**	Trial Statistician	Delegated Statistician
**Name:**	Mahesh Parmar	Andrew Embleton
**Signature:**		
**Date:**		

Contents

1. Background and Design 1
   1.1. Background of the trial 1
   1.2. Trial design 1
   1.3. Study Schema 2
   1.4. Summary of major protocol amendment 3
   1.5. Objectives of the trial 3
   1.6. Randomisation 3
2. Outcome Measures 4
   2.1. Primary outcome measure 4
   2.2. Secondary outcome measures 4
3. Data 4
   3.1. Case Report Forms and variables 4
   3.2. Management of datasets 5
   3.3. Data completion schedule 6
   3.4. Data verification 8
4. Definition of Key Terms 8
5. Sample Size 9
   5.1. Background 9
   5.2. Sample size 9
   5.3. Data maturity 9
6. Analysis Principles 9
7. Final Statistical Report 11
8. Ancillary Studies 13
   8.1. Quality Of Life 13
9. Additional Information in the Clinical Paper 15
10. Signatures of Approval 16

1. Background and Design

1.1. Background of the trial

ICON6 is designed to evaluate the safety and efficacy of platinum-based chemotherapy in combination with cediranib in women with platinum-sensitive relapsed ovarian cancer. Cediranib is an oral targeted small molecule inhibitor of a key signalling molecule Vascular Endothelial Growth Factor (VEGF) which is an oral inhibitor of tyrosine kinase (TK) activity and acts through blockade of the TK receptor. Cediranib (AZD2171) will be administered during platinum-based chemotherapy only (concurrent cediranib), or given during chemotherapy and continued as single agent maintenance therapy for up to 18 months (concurrent and maintenance cediranib).

Full details of the background to the trial and its design are presented in the protocol (version 7.0, 11-Nov-2011).

1.2. Trial design

ICON6 is a randomised three-arm, two stage, double-blind, placebo-controlled multicentre Gynaecologic Cancer InterGroup (GCIG) phase III trial.

All patients will receive 6 cycles of platinum-based chemotherapy. Trial drug will be administered for up to 18 months from randomisation or until progression, whichever is sooner. Patients who have not progressed at 18 months from randomisation can continue Trial Drug until progression, if in the opinion of the clinician and the patient there is continuing clinical benefit.

Patients in Arm A (the reference arm) will receive a platinum-based chemotherapy regimen plus a daily oral placebo tablet for the duration of the chemotherapy and up to 18 months from randomisation or until progression.
Patients in Arm B (concurrent cediranib arm) will also receive a platinum-based chemotherapy regimen plus daily oral cediranib during chemotherapy only, and then an oral daily placebo tablet up to 18 months from randomisation or until progression.
Patients in Arm C (concurrent and maintenance cediranib arm) will also receive a platinum-based chemotherapy regimen plus oral cediranib daily during chemotherapy and then continued up to 18 months from randomisation or until progression.

1.3. Study Schema

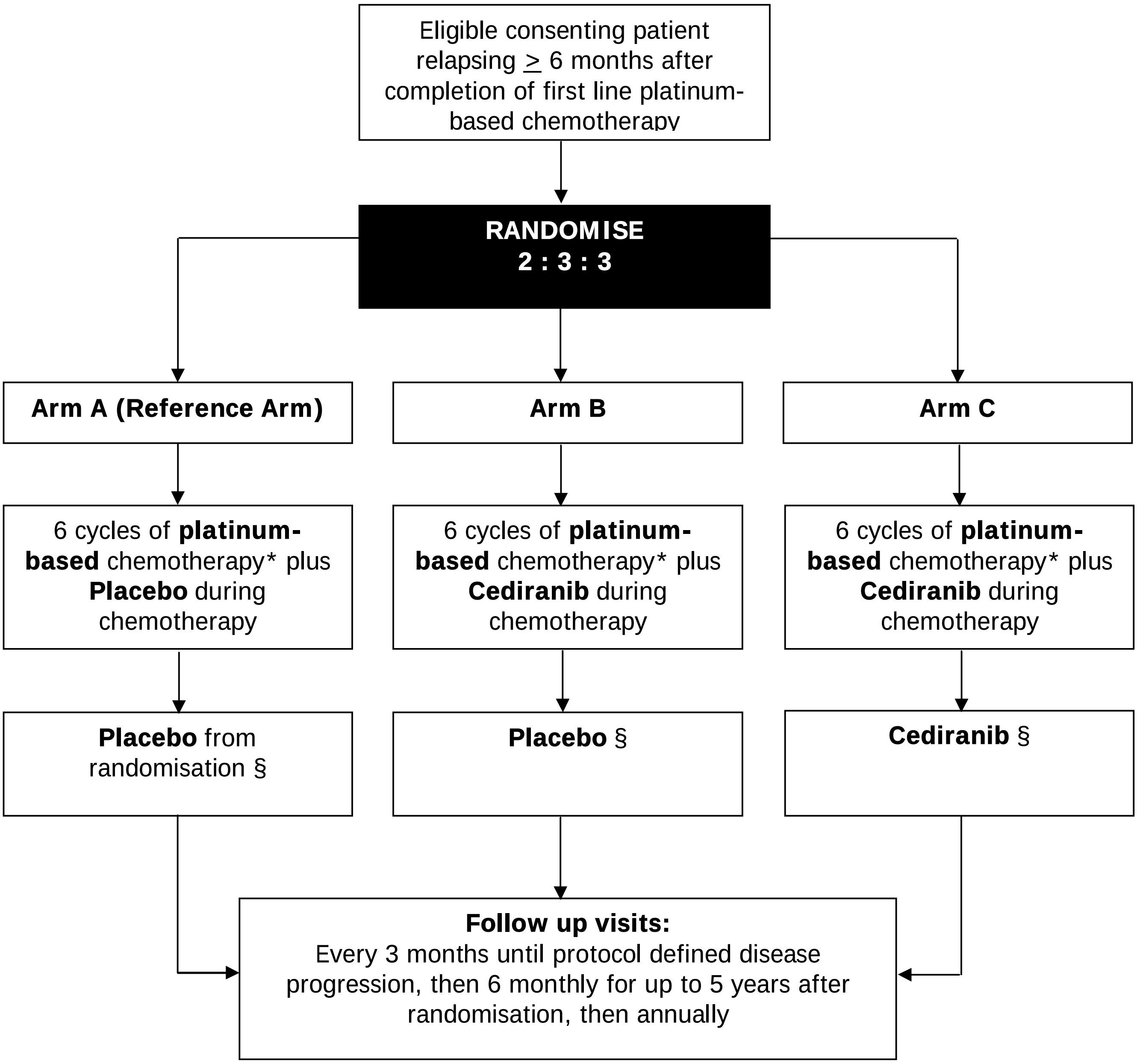

**First Stage:**	Primary outcome measure:	Safety
**Second Stage:**	Primary outcome measure:<br>Secondary outcome measures:	Progression-Free Survival (PFS)<br>Overall Survival (OS) and Toxicity
**Ancillary Studies:**	Quality of Life (QoL)	

* The recommended chemotherapy in ICON6 is carboplatin plus paclitaxel, however treatment with other platinum-based chemotherapy regimens may be permitted, as detailed in section 10.3.
§ Trial Drug (placebo or cediranib) continues for 18 months, or may continue beyond 18 months until disease progression if after discussion with the study Sponsor the clinician feels there is continuing clinical benefit and the patient wishes to continue.

1.4. Summary of major protocol amendment

Following the results of the HORIZON, REGAL and the BR 29 studies (in patients with colorectal cancer, glioblastoma multiforme and non-small-cell lung carcinoma respectively) not meeting their pre-defined goals of clearly improving outcomes, AstraZeneca announced its decision to cease the development and manufacture of cediranib. It was therefore necessary to re-design the ICON6 trial in light of these developments. The decision was made by AstraZeneca that cediranib was not to be developed for marketing; although it was agreed there was a strong rationale for continuing ICON6.

The original second stage analysis with a 2000 patient trial which could compare duration of therapy (B vs. C) was not required. However with revisions to the planned comparisons, looking at Progression Free Survival (PFS) instead of Overall Survival (OS), targeting a larger effect size and reducing the power still provides important information on the effect of an oral angiogenesis inhibitor which acts on a different pathway to bevacizumab. This will be important for future strategies of angiogenesis inhibition with alternatives to bevacizumab which is an expensive, intravenous monoclonal antibody.

The updated sample size calculations, planned independently of any accumulating outcome data in ICON6, are discussed in detail in Section 5. The revision to the trial design, documented in the trial protocol, underwent ethical review ensuring that the trial retains a high quality which will be publishable in a high impact journal.

Further detail of both the original trial design and the revision can be found in the protocol (version 7.0, 11-Nov-2011).

1.5. Objectives of the trial

Primary Aim:

- To determine the efficacy of cediranib when given concurrently with chemotherapy and continued as maintenance treatment, in terms of PFS (Arm A vs. Arm C).

Secondary Aims:

- To determine the efficacy of cediranib when given concurrently with chemotherapy, in terms of PFS (Arm A vs. Arm B).
- To compare the efficacy of cediranib when given concurrently versus concurrent plus maintenance cediranib, in terms of PFS (Arm B vs. Arm C).
- To compare toxicity and quality of life across the 3 treatment arms.
- To compare the efficacy of cediranib when given concurrently with chemotherapy, in terms of OS (across all arms).

1.6. Randomisation

Patients were randomly allocated to one of three treatment Arms A (18 months of placebo), B (six cycles of cediranib, followed by placebo up to 18 months) and C (18 months of cediranib) in a ratio of 2:3:3.

Stratified block randomisation was used with five stratification factors.

- GCIG group
    - UK

    - Canada
    - Australia and New Zealand
    - Spain
- Planned chemotherapy regimen
    - Carboplatin
    - Carboplatin/paclitaxel
    - Carboplatin/gemcitabine
- First line chemotherapy
    - Paclitaxel
    - No paclitaxel
- Duration of relapse free interval
    - 6-12 months
    - >12 months
- Any VEGF inhibitors previously received?
    - Yes
    - No

Randomisation was carried out externally of the MRC CTU by Perceptives IVRS/IWRS system using alternating block sizes of 8 and 16. A randomisation list was generated and kept by Perceptives, with testing of a dummy randomisation list performed by Dr Wendi Qian.

The Project Requirements Specification document contains further details of the randomisation procedure and is stored in the Statistical Master File.

Several rounds of User Acceptance Testing (UAT) of the randomisation system were carried out by MRC CTU staff prior to the study commencing.

2. Outcome Measures

2.1. Primary outcome measure

The primary efficacy measure will be the period of Progression-Free Survival (PFS).

2.2. Secondary outcome measures

The secondary efficacy measures will be

- Overall survival (OS)
- Toxicity
- Quality of Life (QoL)

3. Data

3.1. Case Report Forms and variables

A copy of the Case Report Forms (CRF) and QoL questionnaires are presented in the protocol and the Trial Master File. Details of the variables are presented as in the metadata which forms part of the Trial Master File.

Full details of data collection and timing are described in the trial protocol (version 7.0, 11-Nov-2011).

3.2. Management of datasets

The original plan was for the database to be frozen on 1 October 2012 as the required number of events, 176 progressions (see Section 5), was expected to have been reached by this point in time. This plan was amended, again independently of any accumulating outcome data in ICON6, due to a number of patients continuing voluntarily on cediranib at this time. It was decided to continue follow-up until approximately 5% of patients, or less, remained on cediranib. A number of events over and above the targeted sample size is expected and will only serve to increase the power of the primary comparison.

At the time of analysis:

- The statistician will extract from MACRO a dataset of all data stored in the database. This will act as the frozen dataset. It is the responsibility of the statistician to accurately record the date of freezing and ensure all data is retrieved.
- New data will continue to be entered onto MACRO database.
- If any outstanding data queries are resolved during the analysis that relate to data in the frozen dataset (e.g. problems that are found during analysis or amended CRFs that are returned to CTU), these will be recorded in the Stata do-files and new datasets not downloaded from MACRO again.

3.3. Data completion schedule

Day	Screening /Baseline	Treatment Period Follow Up Visits										
	-28 to -1	Cycle 1	Cycle 2	Cycle 3	Cycle 4	Cycle 5	Cycle 6	Week 21 [21]	Week 27[22]-75[24]	Week 81 [22]	6 weekly Follow-up if continuing on TD beyond Week 81	All further follow up [26]
Informed consent	X											
Demographics, medical history, height	X											
Physical examination including weight	X	X	X	X	X	X	X	X	X	X	X	X
ECG[1]	X											
Vital signs (including BP)[2]	X	X	X	X	X	X	X	X	X	X	X	X
Performance Status	X	X	X	X	X	X	X	X	X	X	X	X
Pregnancy test [3]	X [3]											
Coagulation[4]	X	X [19]	X	X	X	X	X	X	X	X	X	
LVEF[5]	X			X			X		X			
Haematology[6]	X	X [19]	X	X	X	X	X	X	X	X	X	
Biochemistry[7]	X	X [19]	X	X	X	X	X	X	X	X	X	
TSH, T4[8]	X			X			X		X	X	X	
Urinalysis	X	X [20]	X	X	X	X	X		X	X	X	
Platinum-based chemotherapy		X	X	X	X	X	X					
Trial Drug[9]		X	X	X	X	X	X	X	X	X	X	
Tumour assessments[10]	X[10]						X[10]		X[10]	X[10]		X[10]
CA 125	X	X [19]	X	X	X	X	X	X	X	X	X	X
Chest X-ray[11]	X											
Concomitant medication		X	X	X	X	X	X	X	X	X [25]	X [25]	X [25]
Adverse Events	X[18]	X	X	X	X	X	X	X	X	X[23]	X[23]	X
QoL Forms[12]	X	X	X	X	X	X	X	X	X	X	X	X
Bio marker sampling[13, 14]	X	X	X				X					
Tumour Block[14,15]		X										
Blood for DNA [13,16]		X										
Medical Resource Use [17]		X	X	X	X	X	X	X	X	X	X	X

1. Required at baseline, then as clinically indicated.
2. Daily home BP monitoring during first 2 cycles of chemotherapy for patients in stage 1. Home BP monitoring for other patients with hypertension G2 and above. Stage 2 patients will have their blood pressure monitored at baseline and then weekly for the first 3 cycles, by patient self monitoring, clinic or GP as preferred by patient/clinician. If normotensive continue monitoring on day 1 of each cycle. If hypertensive blood pressure should be monitored daily until <grade 2.
3. Pregnancy test only required for fertile women of childbearing potential.
4. Coagulation (either PT, INR or aPTT) required at baseline. INR must be measured repeatedly in patients on warfarin/coumadin.
5. Left Ventricular Ejection Fraction required at baseline only in all patients who have previously received anthracyclines or chest radiotherapy. For these patients LVEF must be repeated after cycle 3, cycle 6 and every 3 months while on trial drug.
6. FBC (plus differential) required during chemotherapy, within 7 days of D1 of cycle 1 and then within 3 days prior to administration of the other chemotherapy cycles.
7. Biochemistry (total bilirubin, creatinine, potassium, ALT or AST, alkaline phosphatase, calcium, phosphate, total protein, albumin). Required, during chemotherapy, within 7 days of D1 of cycle 1 and then within 3 days prior to administration of the other chemotherapy cycles.
8. TSH/T4 required at baseline, cycle 3 and cycle 6, every 12 weeks while on Trial Drug and at each Safety Follow Up visit.
9. Trial Drug oral daily until progression, or 18 months from randomisation. If discontinuing at 18 months, the last 3 week supply will be given at week 75
10. Baseline scan (CT or MRI) to confirm recurrence, then same modality of scan within 3-6 weeks after d1, cycle 6, 12 months, 18 months and to confirm progression. The baseline radiological imaging is required within 4 weeks of randomisation however, a six week window would be considered after discussion with the CI. If the most recent scan was done more than 6 weeks prior to randomisation then it must be repeated.
11. If baseline CXR suggests possible metastases, chest CT must be performed and measurable disease recorded according to RECIST.
12. Quality of Life forms required at baseline (prior to randomisation), on D1 of each cycle of chemotherapy, at each follow up visit while on Trial Drug, then every 3 months until 3 years from randomisation. Patients who progress within 18 months after randomisation should complete QoL forms on day 1 of the first cycle of third line chemotherapy, and at 12 and 24 months from randomisation.
13. Blood for biomarker sampling: depends on the level of participation. Blood sample at progression required for patients participating at Level 3 and above. Further information is given in Appendix 7.
14. No bloods or tumour specimens collected but patient consent for retrospective collection/later blood sampling obtained.
15. Tumour specimens from primary surgery for tissue microarrays.
16. Blood for DNA pharmacogenomics.
17. Not in stage 1.
18. Adverse events collected from consent.
19. Repeat assessments not required if already performed during previous 7 days for screening purposes.
20. Repeat assessment not required if already performed during previous 7 days for screening purposes.
21. All patients still on Trial Drug attend for change.
22. Safety follow up visit to be performed at this time, although if continuing on trial drug beyond week 81, this visit should be performed either 6 weeks after stopping trial drug or prior to commencing other therapies (whichever is first).
23. All Adverse Events ongoing at the time of the safety follow-up visit that are judged to be related to Trial Drug should be followed until they have returned to baseline status, stabilised, or the assessment of causal relationship has been changed. New serious adverse events judged to be related to trial treatment should be reported indefinitely.
24. 6 weekly follow up following completion of chemotherapy while on Trial Drug.
25. Anticancer drugs only.
26. Prior to progression, patients should be seen 6 weekly whilst continuing on trial drug and until end of year 3, every 6 months during years 4 and 5 and yearly thereafter (if no longer taking trial drug). After disease progression is documented, patients should be followed up 6 monthly during the first 5 years after randomisation and yearly thereafter.

3.4. Data verification

Data verification, consistency and range checks will have been performed at the data entry stage by the MRC CTU, as well as checks for missing data (copies can be found in the Trial Master File). Additional range, consistency and missing data checks will be performed, as appropriate, when the analysis is performed (and when the datasets for analysis are constructed).

Given the thorough nature of MRC CTU follow-up procedure it is expected that the issue of missing data will be relatively minimal. High compliance is anticipated with initial data collection as this is close to the time of patient registration. In the event of missing data occurring it is not planned to use imputation methods, although these may be used for sensitivity analyses.

Any problems with trial data will be queried with the Trial Managers or Data Managers as appropriate. If possible, data queries will be resolved, although it is accepted that due to administrative reasons and data availability a small number of problems will continue to exist. This will be minimised.

4. Definition of Key Terms

Term	Definition
Adverse event (AE)	Any event reported to the MRC CTU using an SAE CRF
Case report form (CRF)	Pro forma for collecting data, also known as Clinical Record Form
Date last seen	The date on which the patient was last seen (alive or dead) and will be taken as the maximum of date of randomisation, date of follow-up (except on forms where the patient died), date of death, date of onset or resolution of an adverse event.
Hazard Ratio (HR)	The hazard rate represents the instantaneous event rate for an individual who has already survived to time t.<br>The hazard ratio describes the effect on the hazard rate of treatment allocation as estimated by regression models that treat the log of the HR as a function of a baseline hazard h_0(t) and treatment allocation.
Overall survival (OS)	Time from the date of randomisation to date of death from any cause
Progression-Free survival (PFS)	Time from the date of randomisation to date of first progression or death from any cause, whichever occurs first

Restricted mean survival time (RMST)	The restricted mean survival time, μ(t*) say, of a random variable T is the mean of min(T,t*). It may be evaluated as the area under the survival curve S(t) up to t*

5. Sample Size

5.1. Background

The first stage of the trial was a safety analysis to compare Arm A with the cediranib containing Arms B and C combined, analysed after 33 patients had been randomised and had completed 3 cycles of chemotherapy. This stage was completed in Dec 2009.
The second stage plan was modified in October 2011 following the manufacturer's decision to stop commercial development of cediranib. The revised sample size of 470 patients includes 440 patients randomised after the protocol was amended to a 20mg dose of cediranib. Formal analysis of the second stage includes only the 440 patients randomised after the amendment, the rationale being that the drug was poorly tolerated at the previous dose and compliance was an issue.

5.2. Sample size

Following one year minimum of follow up, the trial was designed to detect a hazard ratio of 0.65 in PFS between Arm A (reference arm) and Arm C, with a 5% significance level and at least 80% power. This requires a total of 176 events in Arms A and C. The prioritisation of the Arm A and C comparison is based on trials using the angiogenesis inhibitor bevacizumab, where the main benefit was observed in maintenance therapy.

5.3. Data maturity

Analysis was originally planned to take place when 176 events had occurred in Arms A and C, expected around Q3 2012. When this time point was reached, a high proportion of patients remained on cediranib and it was decided, independently of accumulating outcome data, to continue follow-up until this proportion had reduced to approximately 5%.

6. Analysis Principles

Descriptive statistics will be reported by randomised group and overall. Percentages will be of non-missing values, with the number (%) of non-missing values given if data are not complete. Percentages will be reported to 0 decimal places, unless <0.5% when they will be given to one decimal place. P-values will be given to 2 significant figures. All statistical tests will be 2-sided.

Time-to-event data will be presented using Kaplan-Meier plots, which may be truncated when there are approximately 10 patients at risk in order to avoid over-interpretation. For the outcome measure of progression-free survival patients who have not progressed will be censored on the date last seen. The difference between randomised arms will be tested using a log rank test. The number of events observed and the log-rank expected number of events will be presented.

The primary analysis method will use the approach outlined by Royston P & Parmar MKB (Statistics in Medicine 2011; 30: 2409-2421) to ensure a valid analysis in the case of non-proportional hazards being observed.

- The treatment effect will be assessed using a log-rank test.
- The proportional hazards assumption will be assessed using a test of non-zero slope in a generalized linear regression of the scaled Schoenfeld residuals on functions of time developed by Therneau and Grambsch to assess whether proportional hazards have been observed.
- If there is evidence of non-proportional hazards at the 5% significance level, a flexible parametric hazards model will be fitted with three degrees of freedom for the baseline distribution and one degree of freedom for the time-dependent treatment effect. The main outcome measure will be the difference in restricted mean survival time (RMST) between each experimental arm and the control arm, with 95% confidence interval, estimated from the flexible parametric model at a pre-specified time of interest (t*) of two years. The choice of t* should cover the clinical period of interest and be reasonably close to the last observed event time. The value of 2 years was chosen according to Figure 1 and the expected follow up time of patients.
- If there is no evidence of non-proportional hazards then a hazard ratio with a 95% 2-sided confidence interval will be presented.

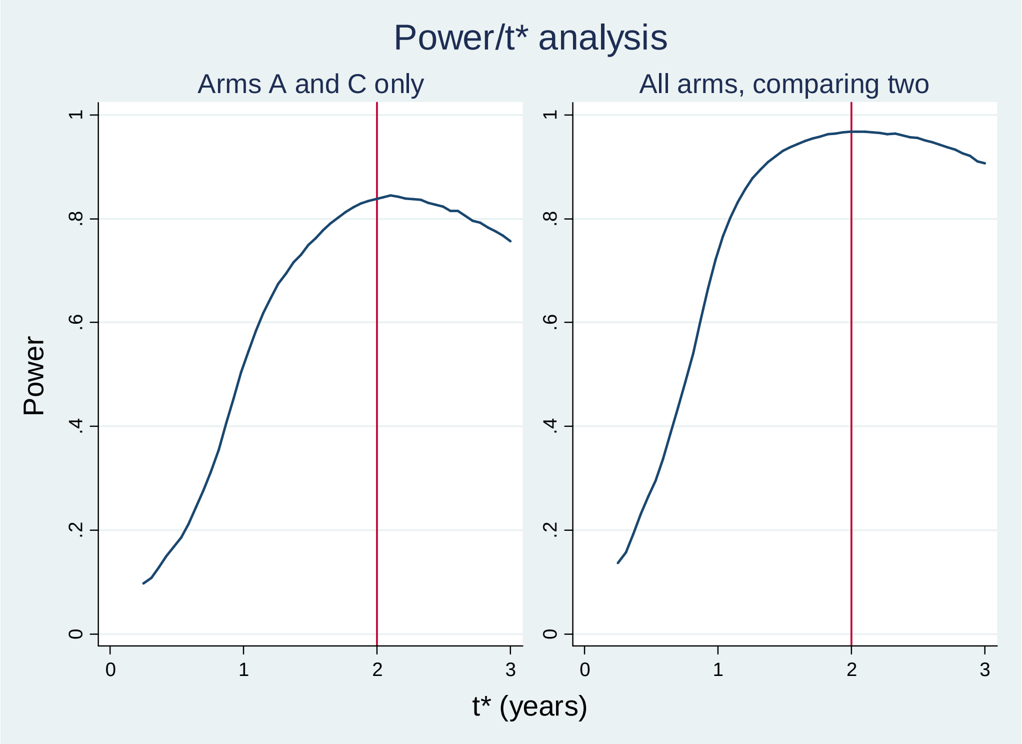

Figure 1: Analysis power vs. candidate values of t*

The analysis approach is illustrated in Figure 2.

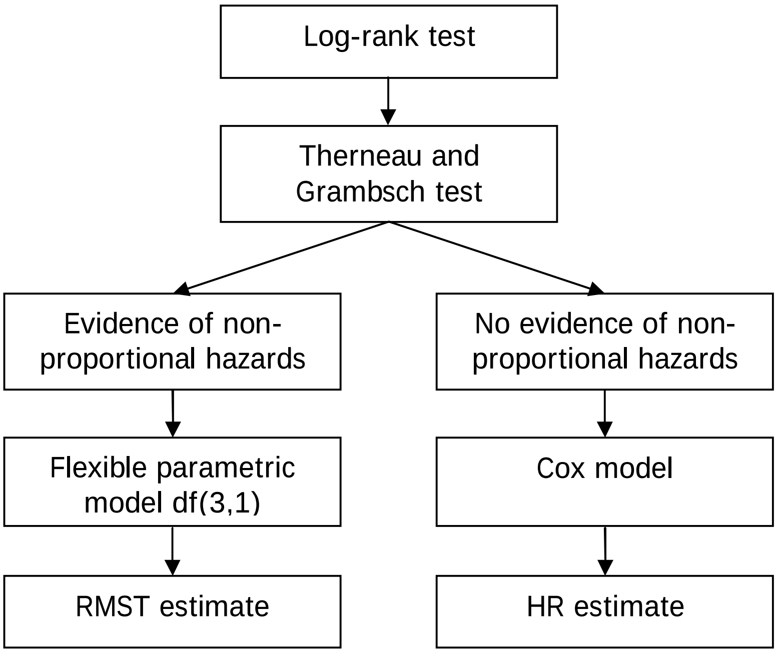

Figure 2: Analysis methods choice and outcomes

Median follow-up time will be calculated. Firstly, using a Kaplan-Meier approach, taking date last seen (if alive) to be an event and death or progression as the time of censorship. The median time will be obtained for each group from life tables. Secondly, in patients who had not progressed, the median time to last follow-up will be calculated using standard summary statistics.

To assess whether any treatment is more or less effective in pre-defined subgroups, chi-square tests for heterogeneity or, when appropriate, trend will be performed. Subgroup analysis will be performed in an exploratory manner keeping the summary statistic consistent with that used in the primary analysis. Potential subgroups are patients with ascites, relapse before/after 12 months, baseline performance status and patients previously having VEGF inhibitors. Forest plots will be presented to visually summarize the consistency of an effect over the subgroups.

7. Final Statistical Report

The results of the analyses will be reported following the principle of the ICH E3 guidelines on the Structure and Content of Clinical Study Reports.

All tables will be presented overall and by arm, where appropriate. Categorical measure will be summarised by counts and relative percentages, and continuous variables will be summarised using the appropriate average measure (mean/median) and measure of spread (standard deviation/inter quartile range/range).

The following serve as an outline for the final reporting of the trial and may be amended:

Enrolment and eligibility
- Eligibility
- Reasons for ineligibilities

Accrual
- Per time period
- Cumulative

Form return rates

Pre treatment
- Age
- Enrolment by origin of carcinoma
- Histological type of carcinoma
- Tumour grade
- First line treatment
- Time since end of first line
  - 6-12 months
  - >12 months
- Previous VEGF
- Planned chemotherapy regimen
- Height (cm), weight (kg), BMI (kg/m2)
- Enrolment by country
- Symptoms

Baseline
- Country
- Centre

Tumour histology
- Time between first histological diagnosis and randomisation (weeks)
- Time between radiological confirmed disease recurrence/progression and randomisation (days)
- Origin of carcinoma
- Histological type
- Grade

Physical examination
- Age
- Blood pressure
  - Diastolic
  - Systolic
  - At least one outside normal range
  - Both within normal range
- Pulse rate
  - Number outside normal range and overall median
- ECOG

Administration of chemotherapy
- Chemotherapy received
- Total number of cycles received
- Reasons for stopping chemotherapy early

Administration of trial drug during chemotherapy
- Any dose omitted
- One dose reduction
- No dose reduction and trial drug stopped
- Never started trial drug

Treatment compliance/adherence
- Cycles received
- Number and reason for stopping/modifying treatment by cycle

Safety
- Baseline symptoms
- Worst toxicity experienced during chemotherapy phase (including/excluding alopecia)
- Worst toxicity experienced during maintenance phase
- Grade 3/4 toxicity during maintenance phase by cycle
- Hypertension experienced during both chemotherapy and maintenance phase by cycle
- Any gastrointestinal perforations
- Breakdown of SAE and SARs

Six chemotherapy cycles
- Vital signs
- Laboratory analyses

Trial events
- Progression
- Death
-

Follow up
- Reason and number lost to follow up
- Participants available at specific time-points
- Duration of follow-up after randomisation

8. Ancillary Studies

8.1. Quality Of Life

A separate Quality of Life analysis plan will be prepared to address the relevant analyses. This section serves to give an overview of an initial draft of these plans.

Focus
The main focus of the quality of life study will be on the symptoms related to epithelial ovarian or primary serous peritoneal carcinoma and their treatments, and questions on overall health and overall quality of life.

Instruments
The questionnaires included were
- EORTC QLQ-C30 (version 3)

- EORTC QLQ-OV28
- EQ-5D

Subscales
The EORTC QLQ-C30 questionnaire includes subscales for Global health status, physical functioning, role functioning, emotional functioning, cognitive functioning, social functioning, fatigue, nausea and vomiting, pain, dyspnoea, insomnia, appetite loss, constipation, diarrhoea, and financial difficulties. The QLQ-OV28 questionnaire includes subscales for abdominal/Gastrointestinal, peripheral neuropathy, hormonal, body image, attitude to disease/treatment, and chemotherapy side effects.

Timing of QoL assessments
As described in Section 3.3, forms are required at baseline (prior to randomisation), on day 1 of each cycle of chemotherapy, at each follow up visit while on Trial Drug, then every 3 months until 3 years from randomisation. Patients who progress within 18 months after randomisation will complete QoL forms on day 1 of the first cycle of third line chemotherapy, and at 12 and 24 months from randomisation.

Outcome measures
The primary QoL analysis will be a comparison between treatment arms A and C of the global quality of life score at 12 months since baseline.

Each subscale from the QLQ-C30 and OV-28 will be described and compared between arms A vs. B and A vs. C arms at each time point:

Role functioning	Cognitive functioning
Constipation	Appetite loss
Dyspnoea	Diarrhoea
Financial consequences	Sleep disturbance
Other chemotherapy side effects	Peripheral neuropathy
Attitude to disease/treatment	Hormonal
Body image	

Sample size
486 patients were recruited and all patients were asked to complete QoL questionnaires.

Analysis
Analysis principles will follow the wider study analysis as set out in Section 6. As all secondary analyses are exploratory

Specific subscale clinically significant differences will be based upon the parameters of Cocks et al, with a small difference defined by the paper being considered clinically significant.

Missing data/sensitivity analyses
Assumptions about missing data may be tested using sensitivity analyses, such as extreme cases of imputation, in order to assess the robustness of the analyses.

9. Additional Information in the Clinical Paper

The intention is for both the main trial results and the Quality of Life analysis to be incorporated into the same publication. This may take the same form as this document where a high level summary is included (such as global scores alone) with a follow up publication addressing patient's quality of life in further detail.

10. Signatures of Approval

Date: 16-Apr-2013
Version: 1.0

Name Andrew Embleton
Trial Role Delegated Statistician

Signature _______________________ **Date** _______________

Name Adrian Cook
Trial Role Senior Statistician

Signature _______________________ **Date** _______________

Name Prof. Max Parmar
Trial Role Senior Statistician/Unit Director

Signature _______________________ **Date** _______________

Name Prof. Jonathan Ledermann
Trial Role Chief Investigator

Signature _______________________ **Date** _______________
